# Shared and Related Molecular Targets and Actions of Salicylic Acid in Plants and Humans

**DOI:** 10.3390/cells12020219

**Published:** 2023-01-04

**Authors:** Yuanyuan Ding, Baofang Fan, Cheng Zhu, Zhixiang Chen

**Affiliations:** 1 College of Life Sciences, China Jiliang University, Hangzhou 310018, China; 2 Department of Botany and Plant Pathology and Purdue Center for Plant Biology, Purdue University, West Lafayette, IN 47907-2054, USA

**Keywords:** salicylic acid, aspirin, systemic acquired resistance, anti-inflammatory, protein phosphorylation, peroxidases, receptors, molecular targets

## Abstract

Salicylic acid (SA) is a phenolic compound produced by all plants that has an important role in diverse processes of plant growth and stress responses. SA is also the principal metabolite of aspirin and is responsible for many of the anti-inflammatory, cardioprotective and antitumor activities of aspirin. As a result, the number of identified SA targets in both plants and humans is large and continues to increase. These SA targets include catalases/peroxidases, metabolic enzymes, protein kinases and phosphatases, nucleosomal and ribosomal proteins and regulatory and signaling proteins, which mediate the diverse actions of SA in plants and humans. While some of these SA targets and actions are unique to plants or humans, many others are conserved or share striking similarities in the two types of organisms, which underlie a host of common biological processes that are regulated or impacted by SA. In this review, we compare shared and related SA targets and activities to highlight the common nature of actions by SA as a hormone in plants versus a therapeutic agent in humans. The cross examination of SA targets and activities can help identify new actions of SA and better explain their underlying mechanisms in plants and humans.

## 1. Introduction

Salicylic acid (SA; 2-hydroxybenzoic acid) is produced by many prokaryotic and eukaryotic organisms including plants. Plants synthesize SA through two pathways: from cinnamate produced by phenylalanine ammonia lyase (PAL) and from isochorismate (IC) produced by IC synthase (ICS) [1]. In plants, SA is a hormone with regulatory functions [2]. In thermogenic plants, SA triggers heat production by activating alternative respiration to volatilize putrid-smelling compounds to attract pollinating insects [3]. The best-established role of SA is as a defense hormone in plant immune responses [2]. SA can activate defense responses in plants characterized by the induced production of plant-pathogenesis-related (PR) proteins and the enhancement of disease resistance [4]. In resistant plants, pathogen infection often induces SA accumulation, not only in infected parts but also in upper uninfected leaves to establish systemic acquired resistance (SAR) [5,6,7]. Blocking SA accumulation or signaling can make plants hypersusceptible to pathogen infection and unable to establish SAR [8,9]. In addition, SA has a regulatory role in plant responses to a variety of abiotic stresses such as heat, drought, salt and high light [10,11,12]. SA-mediated defense and stress responses are often multilayered, involving not only the activation of defense and tolerance mechanisms, but also the modulation of plant growth through crosstalk with other plant hormones such as auxin [13]. Thus, SA regulates plant growth as well.

In humans, SA has medicinal effects. SA-rich plants have been used in humans for millennia to relieve fever and pain, and the commercial production of synthetic SA in the mid-1800s greatly expanded its clinical use until the appearance of aspirin [14]. Aspirin or acetyl SA is a synthetic SA derivative that retains the medicinal properties of SA with reduced stomach irritation [15]. For more than a century, aspirin has been one of the most widely used drugs in the world. In addition to its medicinal effects on fever, pain and inflammation, aspirin has been found to reduce the risk of stroke, heart attack and certain cancers [16]. Aspirin is rapidly metabolized into SA in the human body [17], and many of the anti-inflammatory, cardioprotective and antitumor activities of aspirin have been attributed to SA [14,16,18]. 

Given its broad and diverse activities in both plants and humans, SA has a large number of molecular targets that mediate its actions in a spectrum of biological processes. In plants, NONEXPRESSOR OF PR GENES (NPR) proteins function as SA receptors and mediate SA-mediated defense-related gene expression and immunity [19,20,21]. Over the past 30 years or so, however, other SA-binding proteins have been identified that mediate other NPR-independent actions by SA in plants [14,22]. In humans, prostaglandin H synthases (also known as cyclooxygenases COX1 and COX2) are the key enzymes that catalyze the conversion of arachidonic acid into prostaglandins, which induce fever, pain and inflammation [23]. Aspirin acetylates and irreversibly inhibits COX1 and COX2, and this mode of action by aspirin has been long believed to underlie its anti-inflammatory activity [23]. However, aspirin is rapidly converted to SA in the human body and SA does not acetylate COX proteins but has many similar pharmacological effects as aspirin [22]. These findings indicate that there are additional targets of SA in humans as well, which has been confirmed by a variety of reported studies. In this review, we discuss major plant and human SA targets and activities to highlight the common or similar actions by SA as a hormone in plants versus a therapeutic agent in humans. The comparative and cross examination of SA targets and activities between plants and humans can help identify new actions of SA and better understand their underlying mechanisms. 

## 2. Catalases, Peroxidases and Prostaglandin H Synthases

The first SA-binding protein (SABP1) isolated in plants was a catalase from tobacco [24,25,26]. SABP1 also binds those biologically active SA analogs capable of activating plant defense responses [26]. By contrast, those SA analogs unable to activate plant defense responses fail to bind the tobacco catalases. SA can bind to SA-binding plant catalases and inhibit their activity to elevate the cellular H_2_O_2_ levels [26], possibly mediating some of the actions by SA in plant defense and stress responses. In a more recent published study, Yuan and coworkers provided extensive evidence that *Arabidopsis* catalase 2 (CAT2) functions as an SA receptor in the SA-mediated inhibition of biosynthesis of both auxin and jasmonic acid (JA) [27]. First, in wild-type plants, catalase activity is inhibited and H_2_O_2_ accumulation is increased after pathogen infection [27]. However, in the SA-deficient *sid2* mutant, there is little pathogen-induced catalase inhibition or the accumulation of H_2_O_2_ [27]. Second, the *cat2* mutation can partially rescue the phenotypes of the enhanced disease susceptibility and compromised inhibition of auxin and JA biosynthesis of the *sid2* mutant [27]. Therefore, the inhibition of CAT2 activity is a key mechanism for the induction of disease resistance by SA. Third, increased H_2_O_2_ from the SA inhibition of CAT2 reduces auxin production during SA-mediated resistance to biotrophic pathogens by increasing the sulfenylation of the IAA biosynthesis enzyme tryptophan synthetase b subunit 1 (TSB1), which inhibits its activity [27]. Active CAT2 also physically interacts with the JA biosynthesis enzymes acyl CoA oxidases 2 and 3 (ACX2/3) to stimulate their activities, probably by actively removing H_2_O_2_ generated from the ACX2-/3-calayzed reaction [27]. The SA binding of CAT2 inhibits its activity and, therefore, suppresses the activity of CAT2 to stimulate ACX2/3 and promote JA biosynthesis [27].

Further analysis has revealed that SA can inhibit not only catalases but also peroxidases by acting as an electron-donating substrate that donates a single electron to catalases, thereby trapping the enzyme in an inactive redox state [28]. By acting as an electron-donating substrate for catalases and peroxidases, SA is also converted into SA radicals (SA^∙^), which have been confirmed experimentally [29,30]. Elevated H_2_O_2_ levels as a result of catalase inhibition are involved not only in defense responses but also in plant growth such as SA-induced adventitious root formation in mung bean seedlings [31]. The peroxidase-mediated generation of SA radicals can lead to the generation of other compounds such as superoxide (O_2_^∙−^) and may contribute to the activation of SA-mediated defense responses. SA triggers two phases of oxidative burst with apoplastic cell wall peroxidases involved in the earlier one and NADPH oxidases responsible for the late one [32]. Cell wall peroxidases mediate SA-induced superoxide generation likely through SA radicals in both H_2_O_2_-dependent and -independent manners [32,33]. A critical role of apoplastic peroxidases in SA-mediated defense against the bacterial pathogen *Pseudomonas syringae* has also been established through genetic analysis in *Arabidopsis* [34]. 

Both catalases and peroxidases are iron-containing enzymes, and it has been suggested that SA inhibits these iron-containing enzymes by chelating the iron [35]. Several lines of evidence, however, argue against this hypothesis. First, as discussed earlier, mechanistic and kinetic analyses of catalases and peroxidases have established that SA inhibits these enzymes by acting as an electron donor that traps the enzymes in an inactive redox state, rather than chelating the heme iron of the enzymes. Second, a number of close SA structural analogs have similar binding constants for iron but differ greatly in their ability to bind to and inhibit catalases [24,25]. Finally, we have discovered that even the catalases from different plants or different catalases from the same plants differ substantially in the binding and sensitivity to SA [36,37].

On the other hand, besides plant catalases and peroxidases, there are iron-containing enzymes from other organisms that are sensitive to SA or close SA analogs. They include the heme enzyme myeloperoxidase in human neutrophils, which produces the powerful oxidant hypochlorous acid (HOCl) from H_2_O_2_ and Cl^−^ by inflammatory cells [38]. A variety of anti-inflammatory drugs including SA are effective inhibitors of myeloperoxidase [39,40]. Human prostaglandin H synthase, the major target of aspirin, is a bifunctional enzyme containing both cyclooxygenase and peroxidase activities [41]. The cyclooxygenase activity of prostaglandin H synthase first converts arachidonic acid to prostaglandin G2, which is then reduced to the corresponding alcohol, prostaglandin H2, by the peroxidase activity of prostaglandin H synthase [41]. Therefore, like catalases and peroxidases, prostaglandin H synthase is also an iron-containing heme enzyme. Aspirin irreversibly acetylates a serine residue in the cyclooxygenase site of prostaglandin H synthase. However, the peroxidase of prostaglandin H synthase can strongly affect the acetylation of prostaglandin H synthase by aspirin. First, the acetylation of prostaglandin H synthase by aspirin occurs only when the heme prosthetic group is bound to prostaglandin H synthase [42]. Second, redox cycling of the peroxidase of prostaglandin H synthase inhibits its acetylation by aspirin [43]. As a result, the efficacy of aspirin is increased in cells with high levels of H_2_O_2_ [43]. Therefore, the acetylation of prostaglandin H synthase is promoted by the catalytic activity of the peroxidase that produces a higher oxidative state of the enzyme. 

SA is a weak inhibitor of purified prostaglandin H synthase in vitro [44]. However, in vivo, SA inhibits prostaglandin H synthase with an IC_50_ of only about 30 μM when the enzyme substrate, arachidonic acid, is absent or present at low concentrations, indicating that SA acts as a competitive inhibitor of prostaglandin H synthase [45,46]. The oxygenation of arachidonic acid by the cyclooxygenase activity of prostaglandin H synthase requires the generation of a tyrosyl radical with its active site. Activation of the active site tyrosine residue to its radical state is, however, initiated by the peroxidase site, involving hydroperoxide and the heme prosthetic group. Further analysis has revealed that the inhibitory action of SA on prostaglandin H synthase is dependent on the peroxidase activity of prostaglandin H synthase, most likely by interfering with the activation of the active site tyrosine residue, which is dependent on electron transfer between the cyclooxygenase and peroxidase active sites [47]. These findings illustrate complex patterns of interaction of SA with catalases/peroxidases that are influenced by their redox state and other conditions such as the in vivo levels of substrates and hydroperoxides. 

## 3. NPRs and NFκB in SA-Mediated Transcriptional Regulation

### 3.1. NPRs in Plants

**Figure 1:**
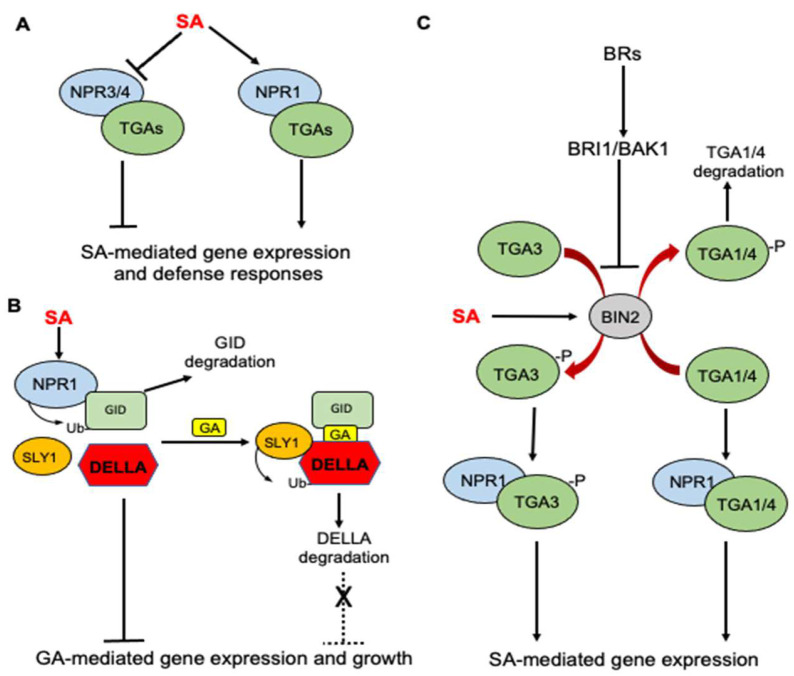
Roles of NPR receptors in SA-regulated defense responses and SA crosstalk with GA and BR in *Arabidopsis*. (**A**) SA binding activates NPR1, which acts as transcription coactivator of TGA transcription factors for SA-mediated gene expression. NPR3 and 4 are transcription repressors but SA can inhibit their repressor activity to release the repression of SA-mediated defense genes. (**B**) SA receptor NPR1 interacts with the GA receptor GID1 to promote its degradation, thereby enhancing the stability of GA repressor DELLA to inhibit GA signaling and plant growth. (**C**) SA activates BIN2 kinase, a negative regulator of BR signaling. Activated BIN2 kinase phosphorylates TGA3 transcription factor and enhances its DNA-binding activity but phosphorylates TGA1/4 transcription factors to inhibit their interaction with NPR1 and decreases their stability.

Like many complex biological processes, SA-induced plant immunity involves the massive reprogramming of plant genes, including the activation of plant defense genes. The SA receptor NPR1 is required for the expression of many SA-induced defense genes [48]. In the absence of SA, NPR1 is present in the cytosol as an oligomer due to the disulfide bridges of its different subunits. Upon SA treatment, the cellular response to induced ROS induces redox change, which leads to the reduction of the disulfide bonds and monomerization of NPR1, which can subsequently translocate to the nucleus to activate defense gene transcription. NPR1 contains an N-terminal BTB/POZ domain, an ankyrin-repeat domain in the middle and a C-terminal disordered domain. NPR1 binds SA through its C-terminal domain [19,49,50], and this SA-binding activity is required for the activation of SA-responsive defense genes by NPR1 [19]. NPR1 contains no DNA-binding activity and functions as a transcription coactivator by binding to a family of DNA-binding TGA transcription factors through the ankyrin-repeat domain. The SA binding of NPR1 induces the folding and docking of the C-terminal SA-binding domain onto ankyrin repeats to activate the transcriptional cofactor activity of NPR [51] (Figure 1A). NPR3 and NPR4 have a similar domain structure as NPR1 but function as negative regulators of immunity by acting as transcriptional repressors of SA-responsive defense genes [19,52] (Figure 1A). NPR3 binds SA with a similar affinity as NPR1, but NPR4 binds SA with a much stronger affinity than NPR1 [19,53]. Under normal growth conditions, when SA levels are low, NPR3 and NPR4 repress defense gene activation. Upon pathogen infection, increased SA binding inhibits the transcriptional repressor activities of NPR3 and NPR4 to release the repression of the SA-responsive target genes (Figure 1A). Therefore, the presence of two types of SA receptors with opposite functions is required for the tight regulation of SA-induced defense responses at different SA levels [54].

There is an extensive crosstalk between SA and other plant hormones for the cooperative regulation of plant defense and balance between defense and growth [13]. Several recent studies have shown that the SA receptor NPR1 and its interacting TGA transcription factors participate in the crosstalk of SA with other plant hormones including gibberellins (GAs) and brassinosteroids (BRs). GAs promote plant growth and SA inhibits GA-mediated gene expression to repress plant growth. GA signaling starts with the binding of GAs to the soluble GID1 receptor, which interact with the DELLA repressor proteins in a GA-dependent manner and targets DELLA protein degradation through the E3 ubiquitin ligase SLY1 [55] (Figure 1B). A recent study discovered that the SA receptor NPR1 interacts with the GA receptor GID1 and functions as an adaptor of ubiquitin E3 ligase to promote GID1 degradation, thereby enhancing the stability of DELLA to inhibit GA signaling and plant growth [56] (Figure 1B).

BR signaling is initiated by BR binding by the plasma-membrane-localized receptor BR-INSENSITIVE-1 (BRI1) and its homologs BRI1-LIKE-1 (BRL1) and BRL3. The binding induces an interaction with BRI1-ASSOCIATED-KINASE-1 (BAK1), leading to the transphosphorylation of their kinase domains [57]. The activation of receptor complexes activates a signaling cascade that inhibits the glycogen synthase kinase 3(GSK3)-like kinase BIN2(Figure 1C). BIN2 is a negative regulator of BR signaling by suppressing the accumulation of BRASSINAZOLERESISTANT-1 (BZR1) and BR-INSENSITIVE-EMS-SUPPRESSOR-1 (BES1) transcription factors in the nucleus, which control the expression of BR-regulated genes [57]. The roles of BRs in plant defense and stress responses are complicated. On the one hand, BR-deficient or -insensitive mutants are more tolerant to plant stress than wild-type plants, indicating a negative role of BRs in plant stress responses [58,59,60]. On the other hand, a number of studies have shown that the exogenous application of BRs promotes stress tolerance and disease resistance in plants [61,62,63,64]. The molecular basis for the opposite effects of endogenous versus exogenous BRs on plant stress responses have been recently investigated and implicate the roles of the NPR1 receptor and TGA transcription factors as well. In a recently reported study, Han and colleagues reported that the BIN2 negative regulator of BR signaling plays a positive role in SA signaling and immunity based on its mutant phenotypes [65]. SA stimulates BIN2 kinase activity, which in turn phosphorylates the TGA3 transcription factor to enhance its DNA binding activity, NPR1-TGA3 complex formation and ultimately SA-mediated defense gene expression [65] (Figure 1C). By contrast, Kim and colleagues recently reported that exogenous BR promotes SA responses by inactivating BIN2 [66]. BIN2 functions as a negative regulator of SA-mediated defense responses by physically interacting with and phosphorylating TGA1 and TGA4 transcription factors to suppress their interaction with NPR1 and promote their destabilization [66] (Figure 1C). Thus, the phosphorylation of different TGA transcription factors by BIN2 appears to have different effects on their DNA-binding activity, interaction with NPR1 and stability. 

### 3.2. Nuclear Factor-κB(NF-κB) in Humans

**Figure 2:**
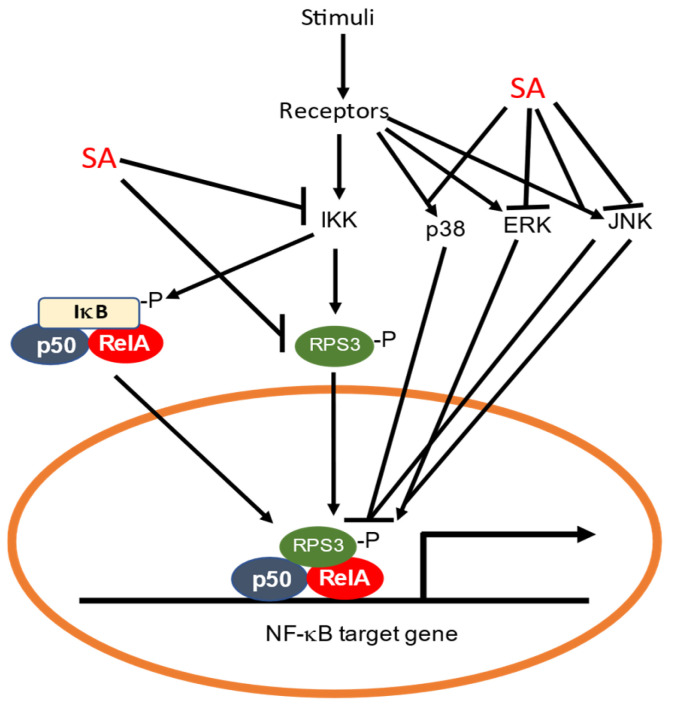
Inhibition of the NF-κB pathway by SA. SA can bind to and inhibit IKK, thereby preventing phosphorylation and degradation of IκB required for activation of NF-κB. Ribosomal protein RPS3 can translocate to the nucleus to activate NF-κB. SA also directly binds to RPS3 to prevent its translocation to the nucleus. The NF-κB pathway can also be inhibited or activated through the p38, ERK and JNK MAPK pathways, which are subjected to inhibition or activation by SA.

NF-κB belongs to a family of inducible transcription factors that regulates a large array of genes involved in multiple aspects of immune and inflammatory responses [67]. The family is composed of five structurally related proteins, NF-κB1/p50, NF-κB2/p52, RelA/p65, RelB and c-Rel. These transcription factors in the forms of Rel/NF-κB homo- or heterodimers bind to a specific DNA element called the κB element at the target gene promoters to regulate their expression [67]. NF-κB proteins are normally sequestered in the cytoplasm by a family of inhibitory proteins called IκB. An important pathway for NF-κB activation in response to diverse stimuli including ligands of various cytokine receptors is the inducible degradation of IκB proteins triggered by their site-specific phosphorylation by the IκB kinase (IKK) complex, which is composed of two catalytic subunits, IKKα and IKKβ, and a regulatory subunit IKKγ [67]. In response to stimuli such as cytokines, growth factors, mitogens, microbial components and stress agents, IKK can be activated and phosphorylate IκB to trigger its ubiquitin degradation by the proteasome system (Figure 2). The degradation of IκB leads to the activation of NF-κB, which then translocates from the cytoplasm to the nucleus to activate the transcription of proinflammatory genes [67].

Both aspirin and SA inhibit the NF-κB pathway [68,69,70,71]. Aspirin and SA specifically inhibit IKKβ activity both in vitro and in vivo [71] (Figure 2). The mechanism of aspirin and SA inhibition is through binding to IKKβ to reduce ATP binding [71]. Thus, the anti-inflammatory properties of aspirin and salicylate are mediated in part by their specific inhibition of IKKβ, which would prevent the activation of NF-κB and the suppression of the genes involved in the pathogenesis of the inflammatory response [71]. Other studies have shown that SA rapidly and persistently activates p38 MAP kinase, which is important for the ability of SA to inhibit tumor necrosis factor (TNF)-induced IκBα phosphorylation and degradation [72,73] (Figure 2). Furthermore, p38 MAP kinase activation appears to play a general role in the inhibition of TNF-induced NF-κB activation [74,75] (Figure 2). 

In a recent study, it was found that SA can also inhibit the NF-kB pathway through ribosomal protein S3 (RPS3), a conserved protein subunit of the 40S ribosome in eukaryotes [76]. Research in animals and human has indicated that RPS3 has extraribosomal roles in DNA repair, cell proliferation, apoptosis and immune responses to pathogens [77,78,79]. For example, RPS3 can translocate to the nucleus upon association with and phosphorylation by IKKβ in response to NF-κB pathway activation [80] (Figure 2). In the nucleus, RPS3 is a NF-κB subunit that helps the selective recruitment of NF-κB to specific promoters and tailors transcriptional responses to specific stimuli [80] (Figure 2). SA binds to RPS3 from human colorectal cancer HT-29 cells [81] (Figure 2). The silencing of RPS3 reduced cyclin-dependent kinase 4 (CDK4) expression and induced G1 phase arrest in human colorectal cancer cells, which were also observed after treatment with SA [81]. Thus, SA negatively regulates the function of RPS3 as a potential mechanism for its protective effects against colorectal cancer. 

## 4. Protein Kinases and Phosphatases

### 4.1. Regulation of Protein Phosphorylation by SA in Plants

**Figure 3:**
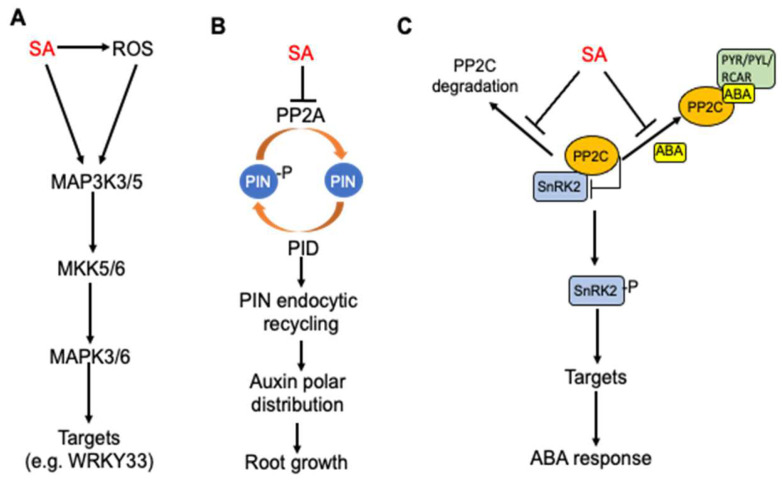
Regulation of protein phosphorylation networks by SA in plants. (**A**) SA-induced MAPK signaling cascade for regulation of defense responses. (**B**) SA inhibition of root growth through inhibition of PP2A, endocytic recycling and polar distribution of auxin. (**C**) SA antagonism of ABA signaling through inhibition of PP2C degradation and ABA-stimulated interaction of PP2C and ABA receptors.

Protein phosphorylation is a reversible post-translational modification of proteins that affect protein stability, subcellular localization and activity. Protein kinases and phosphatases catalyze protein phosphorylation and the dephosphorylation of proteins, thereby working in balance to regulate their substrates’ functions. SA is known to regulate or even directly target the proteins involved in protein phosphorylation that are important for plant defense and stress responses. In tobacco, SA-induced protein kinase (SIPK) is a MAPK important for plant immunity. SIPK is the tobacco ortholog of *Arabidopsis* MPK6, which together with its close homolog MPK3 plays a critical role not only in pathogen-induced defense signaling but also in plant growth and development (Figure 3A). 

A relatively recent study has shown that SA inhibits type 2A protein phosphatases (PP2A) in *Arabidopsis* (Figure 3B). PP2A is a heterotrimer consisting of a scaffold A subunit, a catalytic C subunit and a variable regulatory B subunit with at least 17 isoforms in *Arabidopsis*. Even though they were long considered to be unselective “housekeeping” enzymes, PP2As have been increasingly recognized as specific regulatory proteins involved in signal transduction to activate plant adaptive responses including SA regulation of auxin transport for balancing plant defense and growth during plant–pathogen interactions. SA can affect auxin transport through the regulation of the polar plasma membrane distribution of PIN proteins by altering the phosphorylation of auxin PIN transporter proteins. The reversible phosphorylation of PIN proteins is important for the regulation of their polarity, subcellular dynamics and activity. Several kinases, including PID (PINOID)/WAGs (WAVY ROOT GROWTHs), D6PK/D6PKLs and PAX (PROTEIN KINASE ASSOCIATED WITH BRX) can phosphorylate PIN proteins and multiple phosphatases, including protein phosphatase 2A (PP2A), PP1 and PP6, which can dephosphorylate PIN proteins [82,83,84,85]. SA directly binds to A subunits of PP2A and inhibits the PP2A activity [86] (Figure 3B). The PIN2 auxin transporter is a PP2A target and is consequently hyperphosphorylated in response to SA, leading to changed activity of the auxin efflux transporter and inhibition of auxin transport and auxin-mediated root development, including growth, gravitropic response, and lateral root organogenesis [86]. SA’s action on PP2A, the polar distribution of root auxin and PIN proteins and ultimately root growth are independent of the NPR receptors [86] (Figure 3B).

SA also targets type 2C protein phosphatases (PP2C) to regulate abscisic acid (ABA) signaling [87] (Figure 3C). The most critical components of ABA signaling are ABA receptors, which are members of the pyrabactin resistance 1/PYR1-like regulatory component of the ABA receptor (PYR/PYL/RCAR) protein family [88,89,90]. The binding of ABA by ABA receptors stimulates the tight binding of ABA receptors to PP2C, thereby preventing the PP2C-mediated dephosphorylation of the sucrose nonfermenting 1-related subfamily 2 protein kinase (SnRK2) family members [88] (Figure 3C). As a result, SnRK2 will have increased autophosphorylation and consequently increased kinase activity to transduce the ABA signal by phosphorylating downstream targets [88]. SA directly binds to PP2Cs and can suppress the ABA-enhanced interaction between PP2Cs and ABA receptors (Figure 3C). SA can also suppress the ABA-enhanced degradation of PP2Cs and ABA-induced stabilization of SnRK2s [87] (Figure 3C). All these actions by SA would have negative effects on ABA signaling. Indeed, exogenous SA inhibited ABA-induced gene expression, whereas the SA-deficient *sid2-1* mutant exhibited increased PP2C degradation and increased suppression of seed germination by ABA [87]. These results indicate that SA antagonizes ABA signaling at least in part by directly targeting the critical PP2C components of the ABA signaling complexes.

### 4.2. Protein Kinases as SA Targets in Humans

As discussed earlier, SA can bind and inhibit IKKs to suppress the degradation of IκB and activation of NF-κB. In addition, SA rapidly and persistently activates p38 MAP kinase to inhibit TNF-induced IκBα phosphorylation and degradation [72,73] (Figure 2). P38 MAP kinases are one of three major subfamilies of MAP kinases, with the other two subfamilies being the extracellular-signal regulated kinases (ERK) and the c-jun N-terminal kinase (JNK) [91] (Figure 2). The signaling cascades by the three subfamilies of MAP kinases are important for the regulation of not only inflammation but also for cellular proliferation, differentiation and apoptosis. As such, several key growth factors and proto-oncogenes transduce the signal to promote growth and differentiation through the signaling cascades of these MAP kinases. P38 MAP kinases function as mediators in SA-induced apoptosis. SA inhibits TNT-induced ERK activation [92] (Figure 2). In osteoblasts, TNF plus IFN can induce ERK, IKK and NF-kB activation, as well as COX2 expression. SA inhibits TNF/IFN-induced COX2 expression through the inhibition of ERK and subsequent NF-kB activation in osteoblasts [93]. The effects of SA on JNK activation appear to be cell-type-specific. In normal human F#-4 fibroblasts, SA inhibits the TNF-induced activation of JNK [73] (Figure 2). In COS-1 African green monkey kidney cells and HT-29 human colon adenocarcinoma cells, however, SA treatment leads to a sustained JNK activation. The SA-induced activation of both p38 MAPK and JNK may be relevant to the anti-inflammatory actions of SA [94]. 

**Figure 4:**
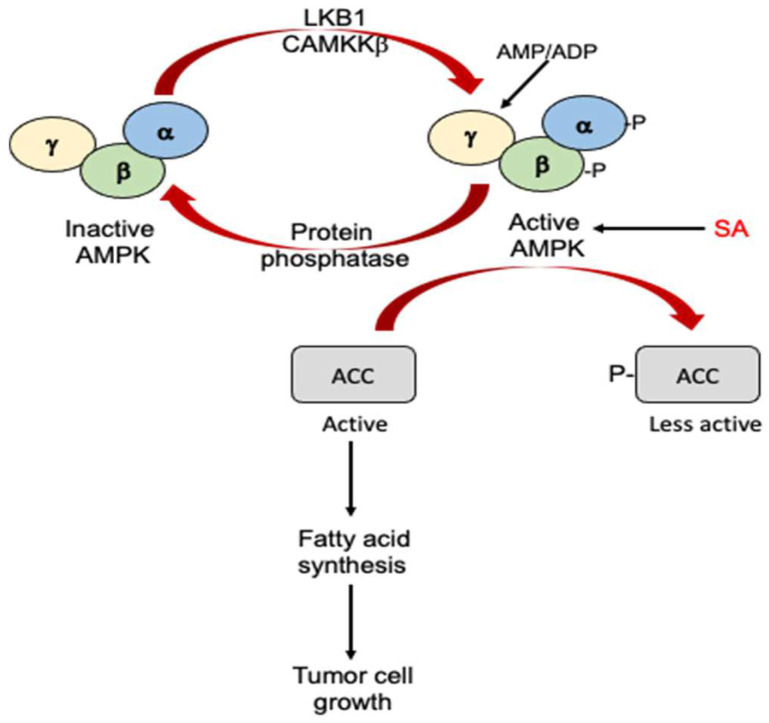
Antitumor activity of SA through activation of AMPK. AMPK is activated through phosphorylation of its α and β subunits by LKB1 and CAMKKβ or through allosteric effects on its γ subunit by AMP/ADP. SA can directly bind to and activate AMPK. Activated AMPK phosphorylates and inactivates ACC. Inactivated ACC suppresses fatty acid synthesis and tumor cell growth.

SA can also activate adenosine monophosphate-activated protein kinase (AMPK), which is a central regulator of cell growth and metabolism conserved throughout eukaryotes [95] (Figure 4). AMPK is a heterotrimeric complex composed of the catalytic α subunit and regulatory β and γ subunits [96,97] (Figure 4). Upon activation under metabolic stress, AMPK phosphorylates targets that suppress adenosine triphosphate (ATP) consuming processes while promoting ATP-generating catabolic pathways. The tumor suppressor protein kinase, LKB1, and the Ca2+-dependent kinase, CaMKKβ, activate AMPK by phosphorylation at Thr172 in the α subunit. The binding of AMP or adenosine diphosphate (ADP) to the γ subunit of AMPK triggers a switch to the active form by promoting phosphorylation and inhibiting dephosphorylation [96,97]. The binding of AMP to a second site can further activate AMPK by inducing further allosteric changes [96,97]. SA activates AMPK by directly binding to the kinase complex, causing the allosteric activation and inhibition of the dephosphorylation of the activating phosphorylation site (Figure 4). Many cancers have high rates of fatty acid synthesis, which is inhibited by AMPK through the phosphorylation of acetyl-CoA carboxylase (ACC) [98] (Figure 4). SA is known to suppress the clonogenic survival of prostate and lung cancer cells and at the same time can increase the phosphorylation of ACC and reduce de novo lipogenesis in an AMPK-dependent manner [98]. These findings strongly suggest that the activation of AMPK and consequent inhibition of lipogenesis by SA is probably important for the anticancer activity of SA.

As a conserved enzyme, PP2A is not only directly targeted by SA in plants but is also subjected to regulation by aspirin in animals. Aspirin displays chemopreventive efficacy in colon cancer, which appear to be associated with the effects of aspirin on the oncogenic Wingless-related integration site (Wnt)/β-catenin pathway activity in colorectal cancer cell lines [99]. Wnt/β-catenin signaling is initiated by the binding of extracellular cysteine-rich WNT glycoproteins to the N-terminal extracellular cysteine-rich domain of the Frizzled family of G-protein receptors [100]. The binding disrupts the destruction complex of β-catenin and triggers the cytoplasmic accumulation of β-catenin, which will then translocate to the nucleus to associate with the T cell factor/lymphoid enhancer factor-1 (TCF/Lef1) transcription complex to activate the transcription of WNT-triggered genes [100]. Wnt/β-catenin signaling is intricately involved in the pathogenesis of several cancers [100]. Aspirin inhibits the activity of this pathway in a dose-dependent manner, as judged by TCF-driven luciferase activity, Wnt target gene expression and the levels and subcellular localization of β-catenin [99]. In addition, aspirin treatment increased phosphorylation and was associated with the inhibition of PP2A enzymatic activity [99]. Using transient transfection with PP2A constructs, the authors further showed that the inhibition of PP2A enzymatic activity was required for the effects of aspirin on the Wnt/β-catenin pathway [99]. The findings revealed a molecular mechanism for the efficacy of aspirin in the chemoprevention of colorectal cancer through the inhibition of PP2A, an important regulator of Wnt/β-catenin pathway activity in these cells.

## 5. High Mobility Group Box (HMGB) proteins

HMGB proteins are nonhistone chromatin-associated nucleoproteins involved in transcriptional regulation, DNA replication and repair, telomere maintenance and nucleosome assembly [101]. In humans, there are four HMGB protein family members, HMGB1, 2, 3 and 4, all of which contain two HMG boxes (A and B) that bind DNA [101]. Human HMGB1 is the most abundant in the four HMGB protein families and can also be actively secreted or passively released by necrotic tissues or stressed cells to the extracellular environment. The extracellular HMGB1 acts as a damage-associated molecular pattern (DAMP) and can bind with several plasma membrane-localized pattern-recognition receptors (PRRs) to mediate cell proliferation, division, migration and differentiation [101]. Recently, the role of HMGB1 as an inflammatory cytokine in carcinogenesis has been widely investigated. Interestingly, aspirin and SA can delay mesothelioma growth by inhibiting HMGB1-mediated tumor progression [102]. Furthermore, HMGB1 directly binds SA through the HMG-box domains [103]. SA suppresses both the chemoattractant activity of the fully reduced HMGB1 and HMGB1-induced expression of proinflammatory cytokine genes and cyclooxygenase 2 (COX-2) [103]. An HMGB1 mutant protein for one of the SA-binding sites still contains chemoattractant activity but fails to bind SA and becomes insensitive to SA [103]. Thus, HMGB1 is a pharmacological target of SA whose binding to HMGB1 directly suppresses its proinflammatory activities.

As highly conserved DNA-binding proteins, HMGBs are present in plants as well. In *Arabidopsis*, there are at least 15 genes encoding HMGBs, which can be further divided into four groups based on the structures and numbers of HMG boxes [104,105]. Some of the *Arabidopsis* HMGB proteins appear to function as nucleosomal proteins, such as human HMGB1. While a majority of these HMGB proteins from *Arabidopsis* are present exclusively in the nucleus, several HMGBs including HMGB2, 3 and 4 are also present in the cytoplasm and therefore have access to the extracellular space upon tissue damage just like human HMGB1 [106,107]. Indeed, the infection of *Arabidopsis* plants by the necrotrophic fungal pathogen *Botrytis cinerea* released HMGB3 into the extracellular space (apoplast) [103]. Extracellular HMGB3 from *Arabidopsis* is a plant DAMP that is capable of inducing innate immune responses, including MAPK activation, defense-related gene expression, callose deposition and resistance to *B. cinerea* through the receptor-like kinases BAK1 and BKK1 [103]. Importantly, like human HMGB1, *Arabidopsis* HMGB3 binds SA, and this binding inhibits the DAMP activity of HMGB3. A mutant HMGB3 protein unable to bind SA still has its DAMP activity but is no longer sensitive to inhibition by SA for induced immune responses [103]. Thus, HMGB proteins function as conserved DAMPs that are subjected to inhibition by SA through direct binding.

## 6. Glyceraldehyde 3-Phosphoate Dehydrogenase (GAPDH)

**Figure 5:**
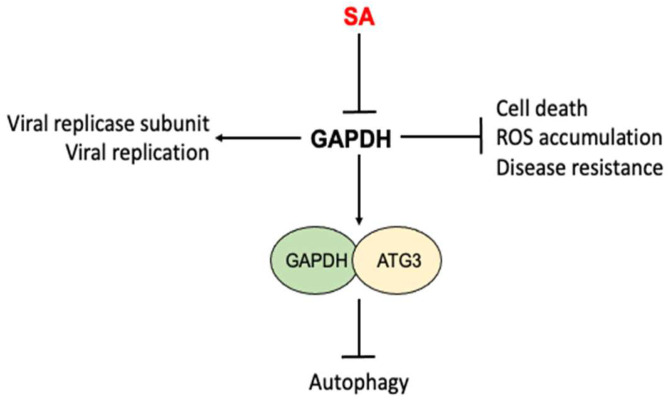
Inhibition of nonmetabolic activities of GAPDH by SA. GAPDHs from both plants and humans participate in a number of nonmetabolic processes including viral replication, cell death, ROS accumulation, disease resistance and autophagy. SA directly binds to GAPDHs and affects the nonmetabolic activities of GAPDH.

SA directly binds both plant and human GAPDH [49,108,109] (Figure 5). GAPDH is an enzyme in glycolysis that catalyzes the transformation of glyceraldehyde 3-phosphate to glycerate-1, 3-biphosphate, accompanied by the production of NADH [110]. In *Arabidopsis*, there are seven GAPDH members, five of which bind SA [49,109]. Among the five SA-binding GAPDHs, three (GAPA-1, GAPA-2 and GAPC-2) are localized in chloroplasts and are involved in the photosynthetic reductive carbon cycle while the other two (GAPC-1 and GAPC-2) are cytosolic proteins [109,111]. In addition to its conserved metabolic function, GAPDH is involved in other nonmetabolic processes. In both plants and animals, it has been reported that GAPDH binds RNAs from a number of viruses [112,113,114,115,116] (Figure 5). Based on a proteomic analysis, GAPDH is one of the co-opted host proteins of the replicase of the *Tomato bushy stunt virus* (TBSV), a single, positive(+)-strand RNA virus that infects a large number of plants [117]. GAPDH preferentially binds the (-) RNA strand of the TBSV, and this binding is required for TBSV replication [115,118]. SA inhibits TBSV replication by inhibiting the binding of cytosolic GAPDH to the negative(-)-strand RNA of the TBSV [109]. Cell biological and genetic approaches have also revealed additional roles of *Arabidopsis* GAPDHs in immune responses to the bacterial pathogen *Pseudomonas syringae* [119] (Figure 5). Knockout mutants for various GAPDH genes displayed enhanced resistance to the bacterial pathogen, accelerated cell death and increased basal ROS accumulation [119]. *Arabidopsis* cytosolic GAPC-1 displayed increased nuclear accumulation in response to pathogen infection [119]. The mutant for GAPA-1 and GAPC-1 also exhibited constitutive autophagy phenotypes under normal growth conditions [119]. Interestingly, *Arabidopsis* cytosolic GAPCs directly interact with autophagy-related protein 3 (ATG3) to negatively regulate autophagy and immunity in *Nicotinana benthamiana* [120] (Figure 5). SA regulates many of these immune- and stress-related responses and it is possible that some of these actions by SA are at least in part mediated through its inhibition of GAPDHs through direct binding (Figure 5). 

In humans, GAPDH also participates in DNA repair, transcription, ROS and cell death/apoptosis [121,122,123,124] (Figure 5). GAPDH plays a critical role in neurodegeneration and is a major suspect in several neurodegeneration diseases including Alzheimer’s, Parkinson’s and Huntington’s diseases. The anti-Parkinson’s drug deprenyl inhibits the nuclear translocation of human GAPDH, which is an early step in cell death [121,123]. Importantly, SA also binds human GAPDH and can suppresses its nuclear translocation and cell death induced by the DNA alkylating agent N-methyl-N’-nitro-N-nitrosoguanidine [108]. 

## 7. Mitochondrial Targets

Mitochondria are powerhouses that generate energy through oxidative phosphorylation, a process of electron movement through the mitochondrial electron transport chain and associated synthesis of ATP and consumption of oxygen [125]. However, mitochondria are also involved in many other important cellular processes, including ion homeostasis, metabolism, cell death and ROS production [126]. In plants, there are two mitochondrial electron flow pathways: the cytochrome respiratory pathway and the alternative respiratory pathway. The two pathways diverge after the ubiquinone pool with electron flow in the alternative pathway directly going to the alternative oxidase, thereby bypassing the two sites of proton gradient formation (compound III and IV). As a result, the energy of electron flow through the alternative pathway is not conserved as ATP, but is released as heat [127]. In floral tissues of some aroid plants including voodoo lily, the capacity of alternative respiration pathway increases drastically, leading to the elevation of the temperature of the tissue by about 9–12 °C above ambient temperature [3]. In thermogenic plants, SA is a signal that triggers heat production by activating alternative respiration to volatilize putrid-smelling compounds to attract pollinating insects [3]. SA can also induce alternative respiration in other plants to affect disease resistance and other biological processes. Interestingly, there have also been studies with strong evidence that SA induces resistance to various viruses in plants in a manner that is dependent on the mitochondrial alternative respiratory pathway [128,129,130,131]. 

SA also has a strong effect on mitochondrial respiration. In a previously published study, we reported that SA at concentrations as low as 20 μM induced the rapid inhibition of both ATP synthesis and respiratory O_2_ uptake in tobacco (*N. tabacum*) cell cultures [132]. Biologically active SA analogs capable of inducing disease resistance also caused the rapid inhibition of mitochondrial respiration, whereas those inactive analogs did not. The SA inhibition of ATP synthesis and respiratory O_2_ uptake was insensitive to the protein synthesis inhibitor cycloheximide, but could be substantially reduced by the antioxidant N-acetylcysteine, suggesting a possible role for ROS [132]. Interestingly, when using NADPH as a substrate, mitochondria isolated from SA-treated tobacco cell cultures were normal in both ATP synthesis and respiratory O_2_ uptake. SA also did not inhibit ATP synthesis or respiratory O_2_ uptake when incubated with isolated mitochondria [132]. These results indicate that the SA-induced inhibition of respiration in tobacco cell cultures may involve components that are not present in isolated mitochondria. However, other groups have shown that SA can inhibit mitochondrial O_2_ uptake when incubated for extended time or acts as an uncoupler when using malate and pyruvate as substrates [133,134]. Further analysis has implicated several mitochondrial components as potential target sites of SA, including complex III and some sites between substrate dehydrogenase and the UQ pool [133,134,135,136]. 

SA binds to and inhibits several enzymes in the critic acid cycle in mitochondria in plants. Aconitase from *N. plumbaginifolia* is sensitive to SA [35]. Interestingly, the inhibition of aconitase by its inhibitor monofluoroacetate causes a rapid and strong increase in the expression of the alternative oxidase gene and alternative respiration in the suspension cells of tobacco [137]. SA also binds to the E2 subunit of the α-ketoglutarate dehydrogenase (KGDE2) enzyme complex from both *Arabidopsis* and tomatoes [138,139]. The KGDE2 activity was reduced almost by 50% in the mitochondria isolated from SA-treated tomato leaves [138]. The silencing of tomato *KGDE2* enhanced tomato resistance to TMV [138]. Thus, the SA inhibition of KGDE2 may play a role in SA-induced antivirus defenses in plants. 

In humans, aspirin and SA also have profound effects on mitochondria, which play a major role in the apoptotic pathway. Many apoptotic signals stimulate the release of cytochrome c from the mitochondria. The released cytochrome c can interact with the apoptotic protease activating factor, Apaf-1, to activate caspase-9, which, in turn, activates other downstream caspases to orchestrate the induction of apoptosis [140,141]. Like in plants, aspirin and SA can uncouple mitochondrial oxidation and induce the mitochondrial permeability transition [142,143,144,145,146]. In liver cells, increased mitochondrial permeability after SA treatment resulted from increased ROS production as a result of the inhibited respiratory chain [147]. SA directly binds to a Fe-S cluster of mitochondrial complex I, the so-called N-2(Fe-S) center, which produces ROS [147]. Mitochondrial permeability transition induction causes an increased release of cytochrome c and apoptotic-inducing factor from mitochondria. SA also interacts with ferrochelatase (FECH) and inhibits its activity [148]. FECH is an enzyme involved in heme biosynthesis in mitochondria. In cultured human cells, SA treatment or FECH knockdown inhibited heme synthesis [148]. These findings indicate that FECH is responsible for the SA-induced inhibition of heme synthesis, which may contribute to its antimitochondrial and anti-inflammatory activity. Furthermore, aspirin and SA inhibit 6-phosphofructo-1-kinase (PFK), the major regulatory enzyme in glycolysis [149], which is metabolically linked with the mitochondrial TC cycle and highly upregulated in tumor cells [150]. Both aspirin and SA inhibit PFK by promoting the dissociation of the active tetramers into inactive dimers [149]. As a result, aspirin and SA decrease glycose consumption and the viability of human breast cancer cells, indicating that one of the mechanisms for the antitumoral effects of aspirin and SA is by directly modulating an important regulatory enzyme in glycolysis [149]. 

## 8. Other Identified SA Targets and Actions

There are other conserved or divergent SA targets that have been identified and characterized in plants and humans. Some of these SA targets have been well characterized, but the mode of action by which SA impacts these targets is not clearly established. Further investigation into these SA targets can provide new insights into the diverse actions of SA as a hormone in plants and as a therapeutic agent in humans.

### 8.1. Carbonic Anhydrase

Tobacco chloroplasts are a β carbonic anhydrase (βCA) with moderate affinity (Kd = 3.7 µM) and are referred to as SABP3 [151]. CAs are conserved enzymes that catalyze the interconversion of CO_2_ and bicarbonate (HCO_3_^−^). In plants, this catalytic activity of CAs is important in a variety of processes including photosynthesis, respiration, stomata movements and lipid biosynthesis [152]. However, plant SABPs/βCAs have additional roles, some of which are closely associated with plant immune responses. First, SABP3 could rescue an oxidative stress-sensitive strain of *Saccharomyces cerevisiae* and, therefore, may have antioxidative properties [151]. Second, SABPs/βCAs are important for resistance to avirulent pathogens in both tobacco and *Arabidopsis*, and this role of *Arabidopsis* SABP3 in immunity requires the nitrosylation of Cys280 [151,153]. Third, SABP3 orthologs from *Arabidopsis* and *Chenopodium quinoa* interact with HCPr, a viral RNA silencing suppressor from *Turnip mosaic virus* (TuMV). HCPro inhibits *Arabidopsis* SABP3 expression and protein accumulation [154]. *Arabidopsis* SABP3 also interacts with NPR1 in the presence of SA [155]. Thus, SABP3//βCAs appear to have roles in both R gene- and SA-mediated defense responses as well as in antiviral RNA silencing. Whether and how SA binding to SABP3//βCAs influences their diverse roles, however, are less understood. 

In humans, there are many described CA isozymes that differ in subcellular localization, catalytic activity and sensitivity to different inhibitors [156]. CAs participate in several important biological processes such as acid–base balance, respiration, carbon dioxide and ion transport, ureagenesis, gluconeogenesis and body fluid generation [156]. Many CA isozymes involved in these processes are important therapeutic targets of disorders including oedema, glaucoma, obesity, cancer, epilepsy and osteoporosis [157,158,159,160]. Interestingly, SA also inhibits human CAI and CAII, the two major CA isozymes in human blood [156]. CAs have esterase activity, which has often been used for assays of CA activity. The esterase activity of human CAII is a carboxylesterase responsible for the short half life of aspirin in the blood by converting it to SA, which subsequently inhibits CAII [161]. Therefore, aspirin is a suicide inhibitor of CAII in humans [161]. 

### 8.2. Thimet Oligopeptidases (TOPs)

TOPs are a family of metallopeptidases found in animals and plants. In animals and humans, TOPs are involved in the degradation of peptides such as bradykinin, neurotensin, angiotensin and AB peptide to help regulate associated physiological processes [162]. *Arabidopsis* contains three TOPs, two of which, TOP1 and TOP2, bind and are inhibited by SA [163]. A genetic and molecular analysis indicated that *Arabidopsis* TOP1 and TOP2 are involved in SA-mediated signaling and in the immune response to avirulent pathogens [163]. TOP1 and TOP2 can form homo- and heterodimers, which was influenced by SA and the reducing agent dithiothreitol [164]. These findings suggest that SA-binding TOP proteins also have biological functions in redox-sensitive cellular processes [164].

### 8.3. Microrchidia (MORC) Proteins

MORC proteins identified in both prokaryotes and eukaryotes contain two hallmark domains, a GHK-type ATPase and an S5 fold [165]. They also possess endonuclease and topoisomerase activities involved in epigenetic gene silencing and DNA modifications [166,167]. SA binds to both plant and human MORCs and suppresses their decatenation but not relaxation activity [167]. In humans, there are five MORC members, and mutations of some of these MORC genes are linked with different cancers or tumor suppression. In *Arabidopsis*, there are seven MORC members. The mutations or silencing of *Arabidopsis* MORC1 and its homologs in other plants affects disease resistance [168,169,170]. The findings that SA binds to and inhibits MORC proteins raise the possibility that SA could also affect cell proliferation and immune responses through effects on epigenetic regulation and DNA modification. 

### 8.4. p300/CREB-Binding Protein (CBP)

P300 and CBP are similar transcription coactivators that interact with several transcription factors to increase the expression of their target genes [171,172]. In humans, CBP and p300 have also been found to be involved in multiple rare chromosomal translocations that are associated with acute myeloid leukemia [173]. SA specifically inhibits CBP and p300 lysine acetyltransferase activity in vitro by direct competition with acetyl-Coenzyme A at the catalytic site. SA also blocked the acetylation of lysine residues on histone and nonhistone proteins in cells. SA could also suppress the growth of p300-dependent leukemia cell lines expressing AML1-ETO fusion protein in vitro and in vivo. These results highlight a novel regulatory mechanism of action by SA for its anti-inflammation and antitumor activities. Plants also contain p300/CBP-like proteins with conserved domains and roles in regulation of gene expression [174,175,176]. It will be of interest to determine whether SA also targets plant p300/CBP to regulate multiple aspects of plant growth, development and stress responses. 

## 9. Summary and Perspectives

In this review, we provided a comprehensive discussion of shared and similar molecular targets and actions of SA in plants and humans. There are other characterized plant- or animal-unique SA targets such as plant SABP2, a methylSA esterase [177,178] and various human neuronal receptors [179,180]. There are also other proteins such as glutathione S-transferases and thioredoxins that bind SA, but the effects of SA binding on their activities and associated biological processes remain to be characterized [49,139]. Therefore, there are a large number of SA targets in both plants and humans that mediate the diverse actions by SA as an important hormone in plants and as a therapeutic agent in humans. Despite the great progress over the past several decades, there are still many important questions about SA targets and actions. First, even though many SA targets have been identified, it remains to be determined whether there are additional unknown targets that are important for the actions of SA. Many of the methods for the detection of SA binding targets rely on traditional in vitro assays, which may fail to identify those targets that recognize SA in a highly sensitive manner. On the other hand, there are other identified SA-binding/sensitive proteins from both plants and humans. For example, a genome-wide screen identified almost 100 SA-binding proteins in human HEK293 cells with roles in multiple biological processes, but the physiological relevance of many of these human SA-binding proteins to the actions of SA remains to be determined [181]. Second, other than the well-characterized prostaglandin H synthases in humans and NPR proteins in plants, we know much less about other SA targets and related actions. Many of these SA targets have important functions in specific cellular and molecular processes, but it is unclear how SA binding affects their activities and associated biological processes. In plants, various SA-binding protein have a substantial spectrum of affinities for SA with their *K*_d_ values spanning from 0.046 to 15.5 μM [22]. SA levels in plants also vary greatly from 0.04 to 10 μg/g of fresh leaf weight depending on plant species and their physiological states [22]. It is, therefore, important to establish whether the physiological SA levels are sufficiently high for the binding affinity of a SA-binding protein to be impacted in the relevant tissue type, subcellular compartment and physiological states. Many SA targets are evolutionarily conserved but have been characterized either only in plants or in animals. For example, SA targets the highly conserved ribosomal protein RPS3, a multifunctional protein important not only for protein synthesis but also in other important cellular processes including DNA repair, cell proliferation and immune responses. It would be of interest to determine whether plant RPS3 proteins are also multifunctional and, if so, whether SA also binds to the conserved proteins and regulates their functions. Third, despite the large number of targets, almost all published studies on SA and aspirin only focus on a single or, at most, a few related targets. This approach, while important for elucidating the individual targets, is not effective for understanding the complex nature of the effects of SA and aspirin. A system biology approach to interrogate the complex biological impacts of SA through a comprehensive analysis and quantification of multiple SA targets and associated pathways can broaden our understanding of the many important biological processes that are subjected to regulation by SA. This knowledge can be further exploited to develop better strategies to improve crop plants and better therapeutic agents for human diseases. 

## Data Availability

Not applicable.
